# Clinical and functional variables can predict general fatigue in patients with acromegaly: an explanatory model approach

**DOI:** 10.20945/2359-3997000000127

**Published:** 2019-04-15

**Authors:** André da Cunha Michalski, Arthur de Sá Ferreira, Leandro Kasuki, Monica R. Gadelha, Agnaldo José Lopes, Fernando Silva Guimarães

**Affiliations:** 1 Centro Universitário Augusto Motta Programa de Pós-Graduação em Ciências da Reabilitação Centro Universitário Augusto Motta Rio de Janeiro RJ Brasil Programa de Pós-Graduação em Ciências da Reabilitação, Centro Universitário Augusto Motta, Rio de Janeiro, RJ, Brasil; 2 Universidade Federal do Rio de Janeiro Faculdade de Medicina Hospital Universitário Clementino Fraga Filho Universidade Federal do Rio de Janeiro Rio de Janeiro RJ Brasil Centro de Pesquisa em Neuroendocrinologia, Serviço de Endocrinologia, Faculdade de Medicina, Hospital Universitário Clementino Fraga Filho, Universidade Federal do Rio de Janeiro (UFRJ), Rio de Janeiro, RJ, Brasil; 3 Instituto Estadual do Cérebro Paulo Niemeyer Secretaria Estadual de Saúde do Rio de Janeiro Rio de Janeiro RJ Brasil Divisão de Neuroendocrinologia, Instituto Estadual do Cérebro Paulo Niemeyer, Secretaria Estadual de Saúde do Rio de Janeiro, Rio de Janeiro, RJ, Brasil; 4 Universidade Federal do Rio de Janeiro Departamento de Fisioterapia Universidade Federal do Rio de Janeiro Rio de Janeiro RJ Brasil Departamento de Fisioterapia, Universidade Federal do Rio de Janeiro (UFRJ), Rio de Janeiro, RJ, Brasil

**Keywords:** Acromegaly, pulse wave analysis, fatigue, exercise tests, muscle strength

## Abstract

**Objective:**

To evaluate whether hormonal profile, arterial function, and physical capacity are predictors of fatigue in patients with acromegaly. Subjects and methods: This is a cross-sectional study including 23 patients. The subjects underwent a Modified Fatigue Impact Scale (MFIS) assessment; serum growth hormones (GH) and IGF-1 measurements; pulse wave analysis comprising pulse wave velocity (PWV), arterial compliance (AC), and the reflection index (IR_1,2_); dominant upper limb dynamometry (DYN); and the six-minute walking distance test (6MWT). Multiple linear regression models were used to identify predictors for MFIS. The coefficient of determination R^2^ was used to assess the quality of the models’ fit. The best model was further analyzed using a calibration plot and a limits of agreement (LOA) plot.

**Results:**

The mean ± SD values for the participants’ age, MFIS, PWV, AC, IR_1,2_, DYN, and the distance in the 6MWT were 49.4 ± 11.2 years, 31.2 ± 18.9 score, 10.19 ± 2.34 m/s, 1.08 ± 0.46 x10^6^ cm^5^/din, 85.3 ± 29.7%, 33.9 ± 9.3 kgf, and 603.0 ± 106.1 m, respectively. The best predictive model (R^2^ = 0.378, R^2^ adjusted = 0.280, standard error = 16.1, and P = 0.026) comprised the following regression equation: MFIS = 48.85 - (7.913 × IGF-I) + (1.483 × AC) - (23.281 × DYN).

**Conclusion:**

Hormonal, vascular, and functional variables can predict general fatigue in patients with acromegaly.

## INTRODUCTION

Acromegaly is a chronic endocrine disease characterized by excessive production of growth hormones (GH) and insulin-like growth factor type I (IGF-I) (1). The insidious process and the difficulty for health professionals to recognize its clinical characteristics often delay diagnosis. Usually, patients are diagnosed 8 to 10 years after their first clinical manifestations, and the disease most commonly occurs in patients between 30 and 50 years of age. There is no significant propensity to sex (2). Clinical manifestations may progress gradually to facial and extremity deformities as well as musculoskeletal, cardiac, respiratory, and metabolic dysfunction and early death. In acromegaly, the normalization of GH and IGF-I levels is associated with clinical improvement (3-7).

Although assessing fatigue in individuals with acromegaly is recommended (8), not using adequate instruments makes it difficult. Fatigue is a subjective symptom, typically defined as extreme and persistent tiredness or physical and mental exhaustion, with characteristics different from those observed in depression or muscle weakness (9,10). It is adequately measured by using a self-report questionnaire in which the individual describes and punctuates the symptoms. One of these instruments is the Modified Fatigue Impact Scale (MFIS), which facilitates the understanding of fatigue and its impact (9).

Despite the physiopathology of general fatigue in acromegaly being unclear (11), its identification assists in diagnosis (8), as it is a common manifestation of the disease (12-15). In the study of Woodhouse and cols. (11), the authors speculated that muscle fiber type, muscle strength, physical capacity, and hormone levels might play a role in the fatigue complaints in patients with GH deficiency or excess GH. However, there are no conclusive studies that have identified the determining factors of fatigue in this population. We hypothesize that GH and IGF-I levels, peripheral arterial function, muscle strength, and functional capacity are associated with fatigue scores among this population. Therefore, this study aims to evaluate whether hormonal profile, arterial function, and physical capacity are predictors of fatigue in patients with acromegaly.

## SUBJECTS AND METHODS

### Study design

This cross-sectional study aimed at investigating the association between some specific characteristics and fatigue in patients with acromegaly. For this purpose, a multivariate analysis was performed using linear regression. A set of arterial function, functional capacity, and hormonal variables were tested as explanatory in linear regression models to predict fatigue (the dependent variable).

### Participants

This study was performed between September 2016 and May 2017 with a cohort of patients with acromegaly. Consecutive subjects aged over 18 years were recruited at the Acromegaly Clinic of the Hospital Universitário Clementino Fraga Filho Federal University of Rio de Janeiro, Brazil. They were included if a diagnosis had been confirmed by an IGF-I level above the age-adjusted upper limit of the normal range and by assessment of the absence of GH suppression < 1.0 ng/mL during the oral glucose tolerance test. After treatment, the disease was considered controlled in the presence of a random GH level below 1.0 ng/mL and of an IGF-I level within the age-adjusted reference range. The disease was considered active when IGF-I levels were above the upper limit of the age-specific range of normality or when GH levels remained above 1.0 ng/mL (16). Patients with any of the following conditions were excluded from the study: without regular clinical follow-up, classification of controlled acromegaly for less than six months, severe joint pain, history of medication change in the last six months, respiratory infection in the last month, unable to perform the proposed tests, and women who were fertile and not using contraceptives.

All participants signed an informed consent form, and the protocol was approved by the Research Ethics Committee of the Augusto Motta University Center under the number CAAE 53678316.9.0000.5235.

### Explanatory (independent) assessments

Noninvasive pulse wave analysis (PWA) was used to assess the pulse wave velocity (PWV), arterial compliance (AC), and reflection index (IR_1,2_). The brachial and radial arterial pulse wave signals were captured through the AFA system (17) for PWV calculation, whereas the same pressure pulse signals were used to determine AC using a four-element Windkessel model. The system consisted of piezoelectric transducers fastened with Velcro straps and connected to a preamplifier. This circuit was coupled to a USB-6009 14-bit model acquisition board (National Instruments, Dallas, TX, USA), which was connected to a computer. The sampling rate was 1.0 kHz per channel. The signals were recorded and processed using a program developed in LabVIEW (National Instruments, Dallas, TX, USA) version 8.0 for Windows (Microsoft Corporation, Seattle, WA, USA).

The isometric dominant upper limb dynamometry (DYN) was measured using a handheld hydraulic dynamometer (model SH5001, SAEHAN Corporation, Yangdeok-Dong, Masan, South Korea) according to the American Society of Hand Therapists’ (ASHT) recommendations. Subjects sat comfortably with shoulders slightly adducted, elbows flexed at 90°, and forearms and wrists in neutral positions (18). Three maximal attempts were alternately made in each arm with a contraction duration of three seconds and a rest period of 60 seconds between each trial. The best results were recorded for analysis. The predicted value for manual grip strength was set according to Neves and cols. (19) equation for the Brazilian population.

The six-minute walking test (6MWT) followed the American Thoracic Society’s recommendations (20). It was carried out in a level, 50-meter corridor. The total distance (6MWD), dyspnea (as measured by the BORG scale), heart rate (HR), and oxygen saturation were recorded (20). The predicted distance values were calculated according to Britto and cols.’s (21) equation for the Brazilian population.

### Outcome (dependent) assessments

General fatigue was recorded using the MFIS in which the total score ranges from 0 to 84 (9). Twenty-one questions were divided into three domains: cognitive, physical, and psychosocial function. The lower the score, the lower the fatigue, and a cutoff score of 38 discriminated fatigued from nonfatigued individuals (22).

### Statistics

Descriptive analyses were performed using mean ± SD or frequency (absolute, %) depending on the variable type. Several models were tested with laboratory (IGF-I), vascular (PWV, AC, and IR_1,2_), and functional (DYN and 6MWT) variables used as predictors of MFIS. A forward stepwise method was applied using the determination coefficients (R^2^) value as an estimate of linear model fit. All analyses were performed using SigmaStat 3.1 (SYSTAT Software Inc., Point Richmond, CA, USA). Calibration was verified by an assessment of the calibration plot (measured vs. predicted along with regression lines showing the slope and intercept) and the limits of agreement (LOA) plot (23).

## RESULTS

### Sample characteristics

Of the 27 recruited patients, four were excluded because of outdated IGF-I recordings (n = 3) and low quality of the PWA signal (n = 1). Seventeen presented with active disease, and six presented with controlled disease. The sample included 14 women and nine men, with a mean age of 49.4 ± 11.2 years. The explanatory (independent) variables, such as IGF-I, DYN, and 6MWD, which have predicted values, were normalized by obtained-predicted ratio. PWV, AC, and IR_1,2_ do not have predicted values. The general characteristics of the patients are shown in Table 1, and the univariate correlations between MFIS and the predictors are shown in Table 2.

**Table 1:** Patients’ characteristics

Variables	N = 23
Anthropometric Data	
Body mass (kg)	89.0 ± 12.5
Body height (m)	1.67 ± 0.09
Laboratory Data	
GH (ng/mL)	8.44 ± 19.39
IGF-I (ng/mL)	412.39 ± 295.46
IGF%	1.60 ± 0.98
Pulse Wave Analysis	
PWV (m/s)	10.19 ± 2.34
AC (x 10^6^ cm^5^/dina)	1.08 ± 0.46
IR_1,2_ (%)	85.3 ± 29.7
Physical Capacity	
DYN (kgf)	33.9 ± 9.3
DYN_O_/DYN_P_	0.91 ± 11.24
DTC6M (m)	603.0 ± 106.1
6MWD_O_/6MWD_P_	1.11 ± 0.50
Fatigue	
MFIS	31.2 ± 18.9

Results are presented as mean ± standard deviation. GH: growth hormone; IGF-I: insulin-like growth factor type I at the onset of the study; IGF%: percentage of IGF above the normality threshold; PWV: pulse wave velocity; AC: arterial compliance; IR_1,2_: reflection index; DYN: dominant upper limb dynamometry; DYN_O_/DYN_P_: predicted-obtained dominant upper limb dynamometry ratio; 6MWD: six-minute walk test distance; 6MWD_O_/6MWD_P_: obtained-predicted six-minute walk test distance ratio; MFIS: modified fatigue impact scale.

**Table 2:** Univariate correlations between MFIS and the predictors

Independent variable	r	P-value
IGF%	-0.492	0.0171
AC	0.458	0.0280
6MWD_O_/6MWD_P_	0.143	0.515
PWV	-0.115	0.602
DYN_O_/DYN_P_	-0.0698	0.752
IR_1,2_	-0.00809	0.971

GF%: percentage of IGF above the normality threshold; PWV: pulse wave velocity; AC: arterial compliance; IR_1,2_: reflection index; DYN_O_/DYN_P_: obtained-predicted dominant upper limb dynamometry ratio; 6MWD_O_/6MWD_P_: obtained-predicted six-minute walk test distance ratio.

### The predictive model

The model for MFIS was evaluated using hormonal variables (IGF%), peripheral vascular integrity (PWV, AC, or IR_1,2_), and physical capacity (DYN_O_/DYN_P_ or 6MWD_O_/6MWD_P_) (Table 3). Six multivariate linear regression models for general fatigue (MFIS) were tested (Table 3). The predictive model with the best fit for MFIS showed R^2^ = 0.378; R^2^ adjusted = 0.280; standard error estimate = 16.081; and P = 0.026. The regression equation was MFIS = 48.855 - (7.913 × IGF%) + (1.483 × AC) - (23.281 × DYN_O_/DYN_P_).

**Table 3:** Regression coefficients for MFIS prediction in subjects with acromegaly

Independent variable	Acromegaly (n = 23)	p-value
Model 1	R^2^ = 0.378	0.026
IGF%	-7.913 (3.686)	0.045
AC	1.483 (0.789)	0.076
DYN_O_/DYN_P_	-23.281 (24.309)	0.350
Model 2	R^2^ = 0.364	0.032
IGF%	-7.489 (3.706)	0.058
AC	1.400 (0.795)	0.094
6MWD_O_/6MWD_P_	16.121 (23.487)	0.501
Model 3	R^2^ = 0.276	0.098
IGF%	-10.371 (3.887)	0.015
IR_1,2_	-7.730 (12.757)	0.552
DYN_O_/DYN_P_	-20.924 (26.299)	0.436
Model 4	R^2^ = 0.267	0.109
IGF%	-9.744 (3.857)	0.021
PWV	-0.579 (1.621)	0.725
DYN_O_/DYN_P_	-20.308 (26.527)	0.453
Model 5	R^2^ = 0.265	0.111
IGF%	-9.731 (3,870)	0.021
IR_1,2_	-4.980 (13.018)	0.706
6MWD_O_/6MWD_P_	15.038 (25.746)	0.566
Model 6	R^2^ = 0.263	0.115
IGF%	-9.304 (3.840)	0.026
PWV	-0.449 (1.611)	0.784
6MWD_O_/6MWD_P_	17.282 (25.295)	0.503

IGF%: obtained-predicted insulin-like growth factor-like type I ratio; PWV: pulse wave velocity; AC: arterial compliance; IR_1,2_: reflection index; DYN: dominant upper limb dynamometry; DYN_O_/DYN_P_: predicted-obtained dominant upper limb dynamometry ratio; 6MWD: six-minute walking distance; 6MWD_O_/6MWD_P_: obtained-predicted six-minute walking distance ratio.

### Model calibration

**Figure 1:**
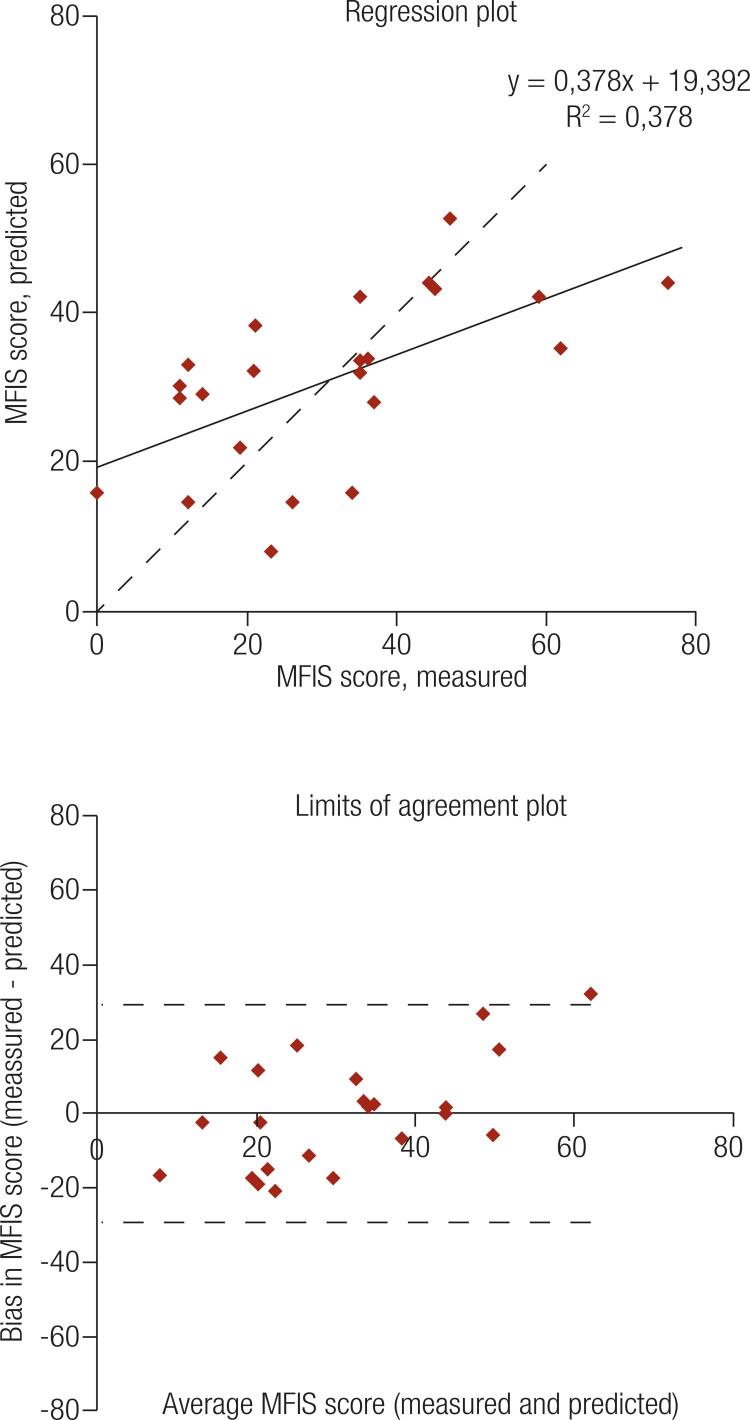
Analysis of the multivariable linear regression model for Modified Fatigue Impact Scale. Top: Calibration plot of the measured vs. predicted Modified Fatigue Impact Scale. Bottom: Limits of agreement plot of the averaged values and the bias (measured-predicted values).

There was no obvious relationship between the bias and the mean (Figure 1, bottom) in the MFIS model. The SD for the bias was a score of 0.0 ± 14.9 (Figure 1, bottom), indicating that the estimate was accurate. The 95% CI for the bias was a score of - 6.5 to 6.5, and the LOA and respective 95% CI for the lower and upper LOA were scores of -29.3 [-40.5; -18.1] and 29.3 [18.1; 40.5], respectively. These intervals are somewhat wide and reflect both the sample size and the moderate variation in the differences.

## DISCUSSION

The present study used the MFIS to develop predictive models of general fatigue in subjects with acromegaly. Along with IGF-I levels, arterial compliance and handgrip dynamometry were important predictors for the model with the best fit. Furthermore, IGF% was an independent predictor in five of the six presented models. This result point to the need for new, longitudinal studies to assess if normalization of IGF-1 during treatment could have a positive impact on physical capacity, AC, and fatigue scores. Conversely, as GH secretion is pulsatile, individuals without the disease may have peaks, and, in turn, patients with acromegaly may present values considered low. Thus, attention is required, as currently there are no reference ranges for baseline GH. IGF-I has no pulsatile secretion or circadian variation that helps identify and characterize the disease (1). The IMMULITE 2000 assay determined reference values for IGF-I according to different ages of the Brazilian population (24).

Several issues remain unanswered regarding the cardiovascular complications and their prognostic implications in acromegaly (25). The increase in the concentration of GH and IGF-I is associated with arterial stiffness (3). In this way, variables of arterial function are important for the early evaluation of these patients. In our study, peripheral arterial function was tested by the PWA (PWV, AC, and IR_1,2_). PWV varies in direct proportion to the tension on the arterial wall (26), whilst IR_1,2_ is the measurement of pulse wave reflection used to assess total vascular resistance (27). AC is an indicator of arterial stiffness along the path traveled by the pulse wave during its propagation (28). Because PWV is associated with cardiovascular diseases in different populations, it is considered a gold standard for assessing arterial stiffness along with known risk factors (29). However, different from AC, PWV and IR_1,2_ were not predictors of MFIS when combined with hormonal profile and physical capacity in our study. It is likely that the high variability of PWV and IR_1,2_ determined a greater dispersion of the data excluding these variables as predictors. The association between vascular impairment and muscle function is well known. Increased risk of peripheral vascular disease progressively restricts perfusion adaptability in skeletal muscle microcirculation (30). It is possible that these vascular impairments play a role in the fiber type changes and muscle dysfunction in these patients, contributing to higher fatigue scores. In this way, many authors have observed that patients with acromegaly present with impaired muscle function and exercise intolerance (12,31,32). As these variables may be associated with general fatigue, Walchan and cols. (31) evaluated a correlation between muscle function variables and Fatigue Impact Scale (FIS). Although they did not find an association with handgrip strength (HGS – isometric dynamometry), a significant correlation was observed between FIS and isokinetic dynamometry of the lower limbs.

In our study, physical capacity was assessed by DYN_O_/DYN_P_ and 6MWD_O_/6MWD_P_. Both were statistically significant when associated with AC, the first being the best predictor of fatigue as verified by the determination coefficient. The upper limb isometric dynamometry is easy to perform and commonly used to represent the general muscle strength. Thus, HGS emerges as a practical alternative for the follow-up of subjects with acromegaly by helping to understand the causes of fatigue as reported by the patients in the clinic. The 6MWT it is a low cost, well-standardized method for assessing functional capacity (20). Its contribution to predicting the fatigue scores found in our study (Table 2: models 2, 5, and 6) is in accord with the results of Thomas and cols. (33) who demonstrated that successful medical treatment of symptomatic patients with acromegaly using octreotide-LAR improved their submaximal and maximal aerobic performance. They also found an association between ventilatory threshold, maximum oxygen uptake, and a reduction in fatigue perception. Although cross-sectional studies are not designed to establish causality relationships, our results suggest that physical function should be assessed in the follow-up of patients with acromegaly. Moreover, it is likely that such subjects might benefit from rehabilitation strategies aiming at improving peripheral muscle function and aerobic capacity, resulting in the improvement of fatigue perception and quality of life.

As the number of predictive (independent) variables in a model depends on the sample size, we could not test models with more than three. Although it can be considered a limitation of this study, we examined many different combinations of hormonal, vascular, and functional variables. Moreover, the Bland-Altman diagram (Figure 1) shows that the estimation was accurate for the best model. Thus, we believe that, in the face of the scarcity of evidence on the causes of fatigue in patients with acromegaly, this study represents an essential contribution to the field.

We conclude that, in patients with acromegaly, general fatigue can be predicted by hormonal profile, peripheral vascular integrity, and functional variables. Future longitudinal studies with a larger sample size are needed to elucidate the pathophysiology of fatigue in such patients and assess if supervised exercise programs positively impact their physical performance and thus result in lower fatigue scores.
